# Prediction of binding hot spot residues by using structural and evolutionary parameters

**DOI:** 10.1590/S1415-47572009000300029

**Published:** 2009-09-01

**Authors:** Roberto Hiroshi Higa, Clésio Luis Tozzi

**Affiliations:** 1 Departamento de Engenharia de Computação e Automação Industrial, Faculdade de Engenharia Elétrica e de Computação, Universidade Estadual de Campinas, Campinas, SPBrazil; 2 Embrapa Informática Agropecuária, Empresa Brasileira de Pesquisa Agropecuária, Campinas, SPBrazil

**Keywords:** hot spots prediction, protein structure, hot spots

## Abstract

In this work, we present a method for predicting hot spot residues by using a set of structural and evolutionary parameters. Unlike previous studies, we use a set of parameters which do not depend on the structure of the protein in complex, so that the predictor can also be used when the interface region is unknown. Despite the fact that no information concerning proteins in complex is used for prediction, the application of the method to a compiled dataset described in the literature achieved a performance of 60.4%, as measured by F-Measure, corresponding to a recall of 78.1% and a precision of 49.5%. This result is higher than those reported by previous studies using the same data set.

## Introduction

Protein-protein interactions play a key role in most biological processes and are of great importance for living cells. Although the principles governing this process are still not fully understood, it is well-known that binding energy is not evenly distributed among interface residues, with a large contribution coming from only a small subset (Moreira *et al.*, 2007). These residues are referred to as binding hot spots.

Recent interest in this protein-protein interface as drug targets (Arkin and Wells, 2004) has highlighted the importance of identifying hot spots systematically. Usually, this is done through site-directed mutagenesis experiments such as the alanine scanning technique (DeLano, 2002). These experiments aim to evaluate the impact in terms of free energy of binding caused by mutations to alanine of specific interface residues. This, however, can demand a significant experimental effort. In this scenario, there is growing interest in cheaper and faster computational hot spot prediction, as they could help biologists focus their experimental efforts only on those interface residues that present the best chance of being hot spots.

Most methods for predicting hot spots rely on physical models to evaluate the impact in terms of free energy of binding due to specific site mutations inside the interface region (Kortemme and Baker, 2002). On the other hand, structure-based methods try to discriminate hot spots from the rest of the interface residues by analyzing their differences through a set of structural and chemical properties. Bogan and Thorn (1998) reported that hot spot residues tend to form clusters near the center of the interface, and are characterized as polar residues protected by a ring of hydrophobic ones that form a structure they call an O-ring. They also analyzed the amino acid preference for being a hot spot and found tryptophan, tyrosine and arginine as those presenting the highest propensities. Another property commonly used for characterizing hot spots is residue conservation. Hot spots have been characterized both as sequentially conserved polar residues (Hu *et al.*, 2000) and as structurally conserved ones (Ma *et al.*, 2003). Li *et al.* (2004) also analyzed the geometric organization of structurally conserved residues concluding that most of hot spots are found in regions characterized by a pocket well-complemented by protruding residues. Other methods include those from Guney *et al.* (2008) that predict hot spots using residue conservation and solvent accessible surface areas - ASA, and the one from Ban *et al.* (2006) that applies a geometric method to predict hot spots by detecting residues located on regions of the interface protected from the periphery.

Only recently, Darnell *et al.* (2007) approached this problem using discriminant analysis, compiling a high quality and non-redundant data set containing interface residues with both types of information: structure and site directed mutagenesis. The best predictor they found involved both structural, chemical and energetic parameters and a combination of classifiers using a simple OR rule. It achieved a performance of 55%, as measured by F-Measure, corresponding to a recall of 72% and a precision of 44%. Using the same data set and a different strategy for combining classifiers, Higa and Tozzi (2008) achieved a slightly higher performance, corresponding to an F-Measure of 56.5%.

In this work we present a method for predicting hot spot residues which rely on a set of structural and evolutionary parameters. Unlike those used by all previously proposed methods, this set of parameters does not depend on the knowledge of protein structure in complex. An SVM classifier (Cristianini and Shawe-Taylor, 2000) with the *a posteriori* probability estimated according to Plat's method (Platt, 2000) and implemented in SVMLib (Chang and Lin, 2001) is used for prediction. Despite the fact that no information concerning proteins in complex is used, the method achieved a performance of 60.4%, measured by F-Measure, corresponding to a Recall of 78.1% and a Precision of 49.5%, which is higher than those previously obtained using the same data set.

## Material and Methods

###  Dataset

We used the data set compiled by Darnell *et al.* (2007). Considering that the number of protein-protein interfaces with organized information characterizing them both structurally and energetically is quite limited, this data set constitutes the most representative one compiled for analyzing hot spot residues. It is composed of interface residues experimentally mutated to alanine and having a reported free energy of binding (ΔΔG) in the AseDB database (Bogan and Thorn, 1998) or in a data set from Kortemme and Baker (2002). The criterion used to define an interface residue is the presence of at least one atom within 4 Å of an atom of the interacting protein. In addition, only proteins whose crystal structure presented a resolution inferior to 3 Å and sequence identity to any other sequence in the data set lower than 35% were considered.

Moreover, we removed from the original data set those residues for which we could not calculate the corresponding conservation property (see below). This corresponds to 15 residues. So, we effectively used a data set containing 233 residues, 24% of them corresponding to hot spot residues. Each residue in the data set was labeled a hot spot if its corresponding ΔΔG reported in AseDB was higher or equal to 2.0 kcal/mol. Otherwise, it was labeled a non-hot spot residue.

### Structural and evolutionary parameters

A set of 43 evolutionary and structural parameters, presented below, were used to characterize an interface residue. Note that all of them are calculated using only the structure of the protein that the residue belongs to.

- Amino acid type (x_1_, x_2_): we used two indexes (Hagerty *et al.*, 1999), derived from the Aaindex database (Kidera *et al.*, 1985), to represent the 20 standard amino acid types. These two indexes summarize a collection of more than 400 indexes describing biochemical properties for each of the 20 standard amino acids. Unlike the equidistant 20-bit code commonly used to encode amino acid type, the more similar two amino acids are, the closer they are in the space defined by (x_1_, x_2_). In particular, the two indexes that we used are strongly correlated to residue size and hydrophobicity on one hand and to residue preference for being in a loop or strand on the other (Hagerty *et al.*, 1999).
- Evolutionary profile (x_3_,..., x_22_): first we used the software Blast (Altschul *et al.*, 1997). As parameters, we used substitution matrix BLOSUM62 and expect value = 0.1, against the Swissprot/Uniprot knowledgebase release 9.6 (Apweiler *et al.*, 2004) in order to find similar protein sequences. Then, sequences in the blast result were filtered according to HSSP threshold (Rost, 1999) to keep only homologue sequences. Two protein sequences in the original data set (Darnell *et al.*, 2007) did not survive this filtering process (at least five homologue sequences). Consequently, in our experiment only 233 interface residues were considered. After that, we used the software ClustalW (Higgins *et al.*, 1994), with substitution matrix series BLOSUM, gapopen = 3.0 and gap ext = 0.1, using the resulting set of homologue sequences to build the final multiple sequence alignment (MSA). Each member of the profile corresponds to the percentage of the amino acid type present in the MSA.
- Conservation score (x_23_): the residue conservation score was calculated using the same MSA used for extracting the evolutionary profile parameters. The residue conservation score corresponds to evolutionary pressure, calculated by using the software rate4site (Pupko *et al.*, 2002). It uses information from the phylogenetic tree built from the MSA and an underlying stochastic process to estimate the residue conservation rates by using the maximum likelihood principle.
- Surface Area and Solvation Energy (x_24_, ..., x_34_): both solvent accessible surface area (SAS) and molecular surface (MS) were calculated by using the program Volbl, included in the software package Alpha Shapes (Liang *et al.*, 1998), considering a probe radius of 1.4 Å and the set of atom radii provided in the package. Also, relative solvent accessible surface area (rSAS) was calculated from the SAS by using the values of SAS for each residue in extended state (Ala-X-Ala), as reported by Ahmed *et al.* (2004). Solvation energy per atom, in cal/mol.Å^2^, was calculated considering four different sets of atomic solvation parameters (ASP) (Eisenberg and McLachlan, 1986; Wesson and Eisenberg, 1992; Fernández-Recio *et al.*, 2004). Additive contribution was assumed such that for each set of ASP, absolute solvation energy per residue was calculated by adding the corresponding solvation energy per atom. In addition, the corresponding solvation energy, weighted per ASA, was also calculated for each set of ASP.
- Geometry (x_35_, ..., x_41_): for describing the geometry of each surface residue, we considered a set of atoms composed of the residue's atoms which were exposed on the surface and all surface atoms as close as 10 Å to any of them. By using the set of coordinates corresponding to each atom in this set, seven geometric parameters were calculated as follows. Gaussian and Mean curvatures were calculated through an osculating quadric, as reported by McIvor and Valkenburg (1997), as well as the corresponding Principal curvatures. From those calculations, Curvedness and Shape Index were also calculated, as proposed by Koenderink (1990). Finally, the Index of Planarity, defined as the reciprocal of the root mean square deviation (rms) of a set of atoms relative to the least square plane through them (Jones and Thornton, 1997), was calculated.
- Dihedral angles (x_42_, x_43_): the software Stride (Frishman and Argos, 1995) was used for calculating ϕ and ψ dihedral angles corresponding to each surface residue.

###  Support vector machines with probabilistic output

In this work, a Support Vector Machine (SVM) was used for classification with the operating point calibrated by using the probabilistic output calculated according to the procedure proposed by Platt (2000) for SVM (Cristianini and Shawe-Taylor, 2000). Considering a training set given by D = {(**x**_*i*_, *y*_*i*_)| **x**_*i*_ ∈ R^n^, *y*_*i*_ ∈ {-1, 1}}, *i* = 1, ..., *m*, where *x*_*i*_ is a *n*-dimensional vector and y_i_ is either -1 or 1, indicating the class to which the object corresponding to *x*_*i*_ belongs to, the most popular formulation for a SVM classifier, known as C-SVC, solves the following quadratic (QP) optimization problem (dual form):


(1)
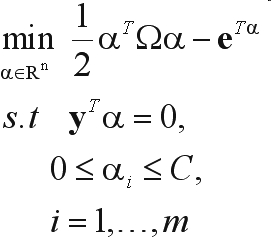


where **e** is a *n*-dimensional vector of ones, α is the *m*-dimensional vector of dual variables, *C* is an upper bound for α_*i*_ value, Ω is a *m* by m positive semi-definite matrix, Ω_*ij*_ = *y*_*i*_*y*_*j*_*K*(**x**_*i*_, **x**_*j*_), and *K*(**x**_*i*_, **x**_*j*_) is a kernel function used for creating non-linear classifiers. In this work, we consider only the radial-basis kernel function, given by:


(2)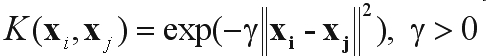.

Usually, a signal function is used to produce a decision function from the SVM unthresholded output:


(3)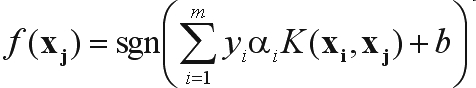 ,

where function f(•) represents the SVM thresholded output, b is a bias term and sgn(•) is the signal function used to produce the SVM thresholded output from its unthresholded one. Objects are classified as belonging to the class corresponding to the label given by f(•).

However, given the practical importance of the *a posteriori* probability in situations where the classifier is making only part of the overall decision process, different methods for estimating the *a posteriori* probabilities for SVM classifiers have been developed (Hastie and Tibshirani, 1998). In particular, Platt (2000) proposed using a post-processing procedure where the SVM unthresholded outputs are mapped into probabilities. For modeling the *a posteriori* probability, a sigmoid function is used:


(4)
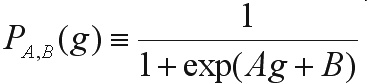


where *g* is the SVM unthresholded output and the parameters *A* and *B* are estimated from the training set by minimizing the corresponding negative log likelihood function:


(5)
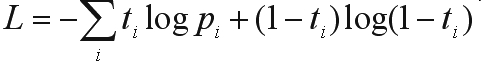


where *t*_*i*_ is the target probability defined as *t*_*i*_ = (*y*_*i*_ + 1)/2 and *p*_*i*_ = *P*(*y*_*i*_ = 1|*g*_*i*_).

###  Performance evaluation

Usually, the performance achieved by a classifier is evaluated by assessing its overall classification error using an independent test set. When the classes involved in the problem have different priors and costs, according to the Bayesian decision theory, the expected overall cost of classification can be used (Duda *et al.*, 2001). This, however, requires the precise specification of the cost of misclassification for each class, which is not always available. For a two-class problem an interesting alternative is to characterize the classifier performance by using ROC analysis (Fawcett, 2006).

A receiver operating characteristic (ROC) curve represents the different tradeoffs between the true positive rate and the false positive rate achieved simultaneously by a classifier, regardless of classes' priors and misclassification costs. In the present context, the ROC curve represents the tradeoff between the rate of hot spot detection and the rate of non-hot spot residues classified as hot spots. Assuming classifiers whose output is a score indicating that an object belongs to the class of interest, each operating point has a corresponding threshold above which objects are classified as belonging to the class of interest. Then, by specifying this threshold, the user is able to specify the operating point most appropriate for his/her application. In addition, the classifier performance can also be summarized through a single scalar, the area under ROC curve (AUC). It represents the probability that the classifier will rank a randomly chosen positive sample higher than a randomly chosen negative sample, and is equivalent to the Wilcoxon test of rank (Hanley and McNeil, 1982). In this work, we use AUC for comparison of different classification models (Linear, Quadratic, Parzen and SVM).

Once an operating point has been chosen, the performance of a classifier for a two-class problem can be assessed by using different performance measures. Among them, we chose Precision, Recall and F-Measure, which assess the classifier performance by focusing on the class of interest, hot spot residues in this case. They are calculated according to the following set of equations:


(6)
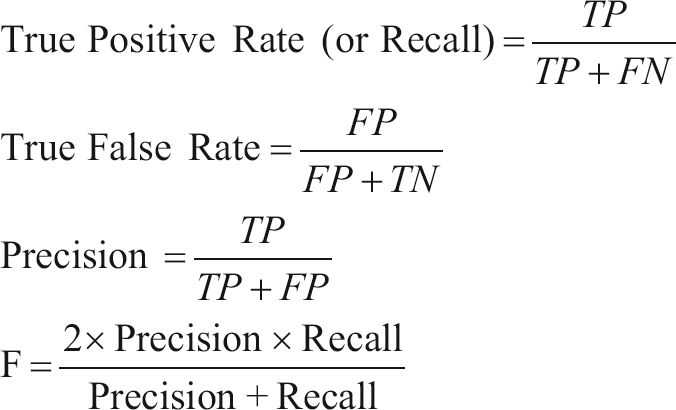


where TP is the number of correctly classified hot spot residues, TN is the number of correctly classified non-hot spot residues, FN is the number of hot spot residues classified as non-hot spot residues and FP is the number of non-hot spot residues classified as hot spots. By using this set of performance measures, we can promptly compare the performance of our method to those reported by previous studies using the same data set (Darnell *et al.*, 2007).

###  Experimental procedure and implementation details

Most parameters used for classification were calculated by using algorithms available as public domain software. For calculating solvation energy and surface shape parameters, Python programming language and Bio.PDB bioPython package (Hamelryck and Manderick, 2003) were used. The Matlab environment 7.0 was used for data analysis and plotting ROC and (Precision, Recall) *vs.* Threshold curves.

The classifier was implemented using the LibSVM software (Chang and Lin, 2001) with the radial basis kernel. For selecting the regularization parameter, *C*, and the kernel parameter, γ, we used a grid search procedure, as suggested in the LibSVM manual. This resulted in the following parameters for the SVM classifiers: *C* = 0.03125 and γ = 0.0078125.

In order to estimate the performance measures (AUC, Precision, Recall and F-Measure) as well as the corresponding graphs (ROC and Precision, Recall, F *vs.* Threshold curves), we used a stratified 5-fold cross-validation procedure. It basically consists of the usual 5-fold cross-validation procedure where the original proportion between classes is maintained in each partition. This procedure was repeated 100 times such that the data set was randomly partitioned each time. We report the average result corresponding to the 100 repetitions. In addition, each time the SVM classifier was trained, we linearly scaled each of the 43 parameters in the training set to the range [-1, 1] and used the same scale mapping to scale the data in the testing set.

## Results and Discussion

###  Classifier performance

Initially, we evaluated three different models for classification - Linear, Quadratic and Parzen (Duda *et al.*, 2001), using AUC as the performance measure. As the best performance was achieved by the Parzen classifier, which is a non-parametric method, we also evaluated the SVM classifier, trying to achieve even higher performance. In fact, the SVM classifier achieved the highest performance among all tested models so that only its results are reported in this section.

**Figure 1:**
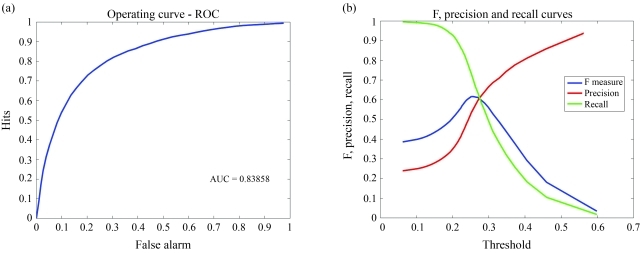
(a) Average operating curve. (b) Average Precision/Recall/F *vs.* Threshold curve.

Figure 1a presents the ROC curve corresponding to the average performance of the SVM classifier considering 100 repetitions of the stratified 5-fold cross-validation. This corresponds to an AUC of 0.8386 (±0.0380) which represents the probability that the classifier ranks a positive sample higher than a negative one, both randomly chosen.

Given that our work is based on the dataset compiled by Darnell *et al.* (2007), it is convenient to compare their results to ours. In their work, Darnell *et al.* (2007) used a decision tree as classifier and achieved a performance of 55%, as measured by the F-measure, corresponding to a Precision of 44% and a Recall of 72%. At this level of Precision, our method achieves a Recall of 83.8% (±5.1), corresponding to an F-Measure of 57.9% (±3.7). In Figure 1b, we present a plot showing how Precision, Recall and F-Measure vary according to the ROC curve operating points, such that the user can choose the most appropriate operating point for his/her application. For instance, if we choose the ROC operating point resulting in the maximum F-Measure value (threshold 0.2427), the classifier achieves a performance of 60.4% (±3.9), as measured by F-Measure, corresponding to a Recall of 78.1% (±5.1) and a Precision of 49.5% (± 4.2). According to the one tail t-test with significance level of 1%, these results are higher than those reported by previous studies using the same dataset (Darnell *et al.*, 2007).

###  Predicting hot spot residues without knowing the interface region

Usually, methods for predicting hot spots (Kortemme and Baker, 2002 and Darnell *et al.*, 2007) assume that the interface region is known, so that their predictions are restricted to interface residues only.

In the present work, we propose a method for predicting hot spots based on a set of structural and evolutionary parameters which do not depend on the availability of the structure of the protein of interest in complex. Only knowledge of the monomer to which the residue belongs is needed. Consequently, the method can be used whether or not the interface region is known. Nevertheless, we emphasize that our method is supposed to detect hot spots among interface residues as defined by Darnell *et al.* (2007).

In order to assess the behavior of the hot spot predictor when the interface region is unknown, we run a simple experiment using the set of residues compiled by Darnell as a training set and all other residues at the surface of the structures considered by Darnell as a testing set. The same regularization and kernel parameters adjusted before were used for training the classifier. The testing set was divided into two groups: one containing residues whose distance to the interacting protein was less than or equal to 7 Å (residues close to the interface region) and another containing the remaining surface residues. The distance between a residue and its interacting protein is defined as the shortest distance between a residue's atom and an interacting protein's atom.

Considering a threshold of 0.2427 to classify a residue as hot spot or non-hot spot, we found that of the 1,023 residues in the group close to the interface region, 384 were predicted as hot spots, corresponding to a rate of positive predictions equal to 37.5%. Similarly, from the 2,155 residues in the group far from the interface region, 632 were predicted as hot spots, corresponding to a rate of positive predictions equal to 29.3%. These numbers suggest that the concentration of positive predictions near the interface region is higher than for distant residues. Moreover, we point out that this difference can become even higher by considering only positive predictions forming clusters at the protein surface (Bogan and Thorn, 1998). Since the interface region is supposed to present a higher probability of hot spot occurrence, the observed higher rate of positive predictions for the group of residues close to the interface region corroborates our *a priori* expectation.

###  Case studies

In order to illustrate the application of the method, we present two examples not included in Darnell's data set. In both cases, the entire data set was used as a training set with regularization and kernel parameters adjusted as before. The threshold of 0.2427 was used for classification. The first example concerns the tetramerization domain of the p53 tumor repressor, a 393 amino acid transcription factor which plays a key role in protecting organisms against cancer (el-Deiry *et al.*, 1992). The p53's tetramerization domain is located at p53's COOH terminal portion and encompasses residues 325-356.

**Figure 2:**
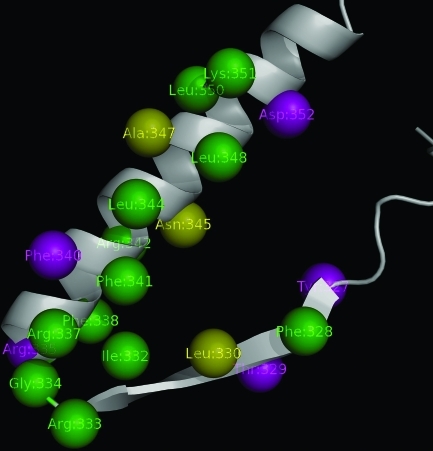
One monomer from the tetramerization domain of the p53 tumor repressor (3sak:A). True positives are indicated in green, false positives in purple and false negatives in yellow.

In an extensive site-directed mutagenesis study, Kato *et al.* (2003) constructed 2,314 mutants representing all possible amino acid substitution caused by a point mutation. By evaluating the level of activity of the mutants, they found that a set of 15 residues at the tetramerization domains were sensitive to inactivation by amino acid substitution: Phe:328, Leu:330, Ile:332, Arg:333, Gly:334, Arg:337, Phe:338, Phe:341, Arg:342, Leu:344, Asn:345, Ala:347, Leu:348, Leu:350 and Lys:351. Considering the 32 residues in the domain, our method identified 12 of the 15 residues reported as sensitive to inactivation, as well as 5 false positives, 3 false negatives and 12 true negatives (Figure 2). This corresponds to an F-Measure of 75% corresponding to a Recall of 80% and a Precision of 70.6%.

The second example is the bone morphogenetic protein-2 (BMP-2), a member of the transforming growth factor-β (TGF-β) with a pivotal role in bone formation and regeneration in adult vertebrates (Reddi, 1998). It signals by binding two types of serine/threonine kinase receptors, classified as type I and type II. Kirsch *et al.* (2000) analyzed interactions of BMP-2 mutants with type I and type II receptor ectodomains and found two different epitopes, each corresponding to a specific type of receptor. One epitope, the strongest one, comprises residues from both monomers (Val:26, Asp:30, Trp:31, Lys:101, Tyr:103 from one monomer and Ile:62, Leu:66, Asn:68, Ser:69, Phe:49, Pro:50, Ala:52 and His:54 from the other) while the other includes residues from only one monomer (Ala:34, His:39, Ser:88, Leu:90 and Leu:100).

**Figure 3:**
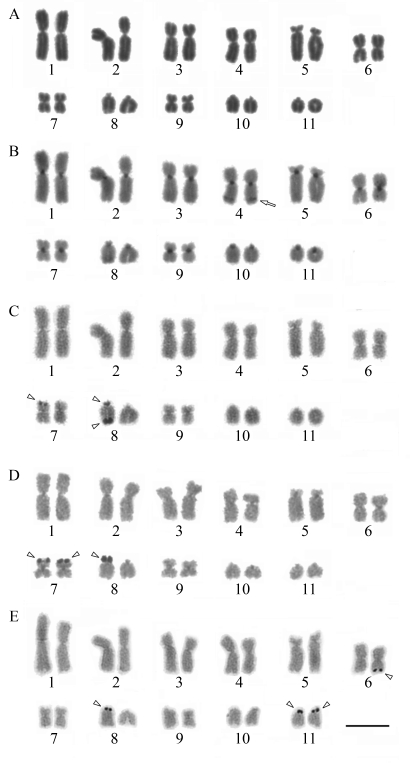
Homodimeric molecule of bone morphogenetic protein-2 (BMP-2) (1es7:A and C).  For the larger epitope (left), true positives are indicated in blue, false positives in red and false negatives in yellow.  Residues in the smaller epitope (right) are indicated in orange.

In this example, we suppose that the epitopes in BMP-2 are unknown and we used our method for evaluating all surface residues of a monomer. The analysis resulted in 29 residues predicted as hot spots, from a total of 101 surface residues. After that, we filtered the set of predicted residues using a sequential window of five adjacent residues so that a positive prediction was kept only if among its two left and two right sequential neighbors at least two of them were also positive predictions. This kind of post-processing is quite common for interface region prediction methods (Yuan *et al.*, 2004; Res *et al.*, 2005). A total of 13 positive predictions survived this filtering process, 5 of them corresponding to residues in the first epitope (true positives). There were also 14 false negatives, 5 false positives and 83 true negatives, resulting in an F-Measure of 34.5% corresponding to a Precision of 50% and a Recall of 26.3%. If only the strongest epitope is considered, it results in an F-Measure of 43.5%, corresponding to a Precision of 50% and a Recall of 38.5%. Even though these levels of coverage (Recall) are quite low, they are typical for interface region prediction methods (Bradford *et al.*, 2006; Neuvirth *et al.*, 2004) and, at a level of Precision of 50%, are considered as satisfactory for locating interface regions (Bradford and Westhead, 2005). Figure 3 summarizes these predictions. While no residue in the second epitope was found, all false positive predictions are close to those in the true positive in the first epitope.

## Concluding Remarks

In this work, we presented a method for predicting hot spot residues within the interface region. By using ROC analysis, we allow the user to choose the most appropriate trade off between true positive and false positive rates, according to his/her specific application. In addition, since the method does not depend on the knowledge of the structure of the protein in complex, it can also be used in situations where the interface region is unknown. Despite these advantages, the performance achieved by the method was also higher than those reported by previous studies using the same dataset.
